# The ABBA study – approach bias modification in bulimia nervosa and binge eating disorder: study protocol for a randomised controlled trial

**DOI:** 10.1186/s13063-016-1596-6

**Published:** 2016-09-26

**Authors:** Timo Brockmeyer, Ulrike Schmidt, Hans-Christoph Friederich

**Affiliations:** 1 Department of General Internal Medicine and Psychosomatics, Heidelberg University Hospital, Heidelberg, Germany; 2 Section of Eating Disorders, Department of Psychological Medicine, Institute of Psychiatry, Psychology and Neuroscience, King’s College London, London, UK; 3 Department of Psychosomatic Medicine and Psychotherapy, Medical Faculty, Heinrich Heine University, Düsseldorf, Germany

**Keywords:** Cognitive bias modification, Approach avoidance training, Computer training, Bulimic eating disorders

## Abstract

**Background:**

The core symptoms of bulimia nervosa (BN) and binge eating disorder (BED) are recurrent episodes of binge eating. Despite negative psychological and physical consequences, BN/BED patients show uncontrollable approach tendencies towards food. This cognitive bias occurs at an early stage of information processing. Cognitive bias modification (CBM) directly targets such biases and has been shown to be effective in treating several mental disorders. In alcohol addiction, automatic action tendencies towards alcohol cues and relapse rates were successfully reduced by a specific form of CBM, termed approach bias modification. Based on these findings and data from a proof-of-concept study in people with high levels of food craving, CBM is considered a promising new treatment approach for BN/BED. Given the similarities between BN/BED and addictive disorders, the rationale for using approach bias modification appears to be particularly strong. The aim of the present study is to examine whether, compared to a sham training, computerised approach bias modification (10 sessions) can reduce binge-eating episodes in BN/BED patients from pre-treatment to follow-up. Additionally, we will investigate whether this CBM programme also reduces global eating disorder psychopathology, trait and cue-elicited food craving, food intake as well as approach and attentional bias towards visual food cues. Treatment acceptance will be determined by attrition rates and responses on a feedback form.

**Methods:**

This is a double-blind, randomised, placebo-controlled, parallel-group superiority trial with two parallel arms. A total of 54 BN/BED patients will be recruited. Approach bias towards food will be retrained by a computer task adopting an implicit learning paradigm. Patients in the control condition (sham) will conduct a similar task but will not be trained to avoid food cues. Methods against bias include public registration, randomisation by a central study office, standardisation of the treatments and blinding of assessors. Furthermore, the session number and duration will be equivalent in the two conditions.

**Discussion:**

This is the first registered randomised controlled trial of approach bias modification in a clinical BN/BED sample. Results from this study will provide an indication of the efficacy of approach bias modification training for BN/BED and the potential mechanisms of action underlying this treatment.

**Trial registration:**

DRKS00010231 (retrospectively registered on 24 March 2016; first version)

## Background

Bulimia nervosa (BN) and binge eating disorder (BED) are common and serious mental disorders with elevated all-cause mortality, comorbidity and high relapse rates [1–4]. They share repeated episodes of binge eating as key characteristics [5]. Several authors have highlighted the addiction-like features in BN and BED [6–9] such as uncontrollable approach tendencies towards binge food despite significant negative psychological and physical consequences.

According to dual-process models, two distinct systems of information processing contribute to the evaluation of food cues and the control of eating behaviour, i.e. an impulsive system that operates rapidly and automatically (i.e. mostly outside of conscious control), and a reflective system that operates slower and more deliberately [10, 11]. The impulsive system evaluates food cues primarily regarding their current emotional and motivational significance. In contrast, the reflective system involves higher-order processes of cognitive control, deliberate decisions and impulse regulation that take long-term consequences into account. It is thought that in addictive disorders, the impulsive system dominates to a large degree the initiation of addictive behaviour, whereas the reflective system lacks power to control this behaviour [11]. Supporting this assumption, bulimic eating disorders such as BN and BED are associated with strong, impulsive responses and poor cognitive control towards food cues, which is considered to contribute to a heightened susceptibility to sensitised cues that trigger action tendencies and lead to binge eating [12]. Hence, even though people with a bulimic eating disorder may be aware of the negative consequences of binge eating, they may still engage in it when food cues are processed in the impulsive system. This is reflected by findings that individuals with bulimic eating disorders show appetitive physical responses towards food cues despite rating them as anxiety-provoking and disgusting [13, 14]. A rapidly growing body of research shows that eating disorders and unhealthy eating behaviour are associated with cognitive biases that unfold their negative effects in the impulsive system during the automatic processing of food cues, particularly by increasing attention and approach tendencies towards food [14–21].

Traditional (talking) psychotherapy programmes may not be best suited to directly modulate such biases that occur at an early stage in information processing and that often operate outside of conscious control [11, 22, 23]. In an effort to create more suitable interventions, researchers have translated findings from basic research and developed cognitive bias modification (CBM) programmes that are considered to provide a way to more directly target such biases. Different CBM programmes have been shown to be effective in treating mental disorders such as depression [24], social phobia [25] and addictive disorders [26–29]. With regards to the latter, several studies have used an approach bias modification training in alcohol-dependent participants and found this to effectively reduce automatic action tendencies towards alcohol cues and relapse rates [27, 29, 30]. Several meta-analyses have demonstrated the efficacy of CBM across different mental disorders [31–35]. Consequently, CBM has also been suggested as a potential new treatment approach for eating disorders [22, 23]. Recently, it was shown that approach bias modification reduces approach bias towards food cues and actual food intake in female student populations [36, 37]. However, it is yet unknown whether such a training is equally effective in clinical samples of patients with BN/BED. In addition, the efficacy may also depend on the use of multiple sessions of training [38].

In a proof-of-concept study [39] we recently evaluated the efficacy of such an approach bias modification training in a sample of people with high levels of food craving and subthreshold bulimic symptoms. Both the targeted approach bias towards food cues and also attentional bias towards food, trait and cue-elicited food craving, as well as more general eating disorder symptoms, were significantly reduced during the training with medium to large effect sizes in this study. The initial approach bias towards visual food cues was not only reduced (as hypothesised) but actually turned into an avoidance bias. Furthermore, all participants completed the intervention and the majority of them perceived the training as effective in altering daily eating habits. Taken together, these highly promising findings suggested that retraining automatic action tendencies in people with bulimic symptoms by means of computerised CBM may be an effective, feasible and acceptable way of reducing cognitive biases, food craving and binge eating. Thus, a randomised controlled trial with BN and BED patients appeared to be the logical next step.

According to the emotional distress hypothesis of binge eating, cognitive biases constitute latent vulnerability factors that become evident when the individual is confronted with a stressor [40]. Consequently, CBM should be most effective in its impact on cognitive biases and subsequent eating behaviour upon exposure to stressful situations. In fact, it has been demonstrated that CBM attenuates the effects of stressors on symptoms of depression and anxiety [41, 42]. Furthermore, there is a common theoretical basis and strong empirical evidence that negative affect (in response to a stressor) is a key trigger of attentional bias towards food and of binge eating [43–46]. Therefore, we applied a vulnerability-stress model for our CBM programme by using stimuli that induce moderate levels of negative affect before cognitive bias training and assessment.

### Aims

The central aim of this randomised controlled pilot trial is to examine whether a specifically tailored, brief (10 sessions), computerised CBM training (approach bias modification versus sham) is able to reduce binge-eating episodes in BN and BED patients (primary efficacy endpoint). More precisely, from pre-treatment to 2-month follow-up, BN and BED patients who receive real CBM are expected to report a greater reduction in the number of objective binge-eating episodes during the previous 2 months than those who receive sham CBM.

Additionally, we will investigate whether this CBM programme reduces global eating disorder psychopathology from pretreatment to follow-up, and trait and cue-elicited food craving, food intake and approach and attentional bias towards visual food stimuli in BN and BED patients from pre- to post-treatment. Finally, we will assess treatment acceptance by attrition rates and by means of a specifically designed feedback form. Specifically, as compared to patients in the sham CBM condition, patients in the real CBM condition are expected to show (1) decreased global eating disorder psychopathology from pre-treatment to follow-up, (2) decreased approach and attentional bias towards food cues, (3) decreased state food craving after cue exposure, (4) decreased trait food craving as well as (5) reduced food intake in a bogus taste test from pre- to post-treatment. In an exploratory analysis we will examine the patients’ acceptance of this specific CBM intervention, taking dropout rates and scores on a feedback form into account (treatment acceptance).

## Methods

### Design

**Fig. 1:**
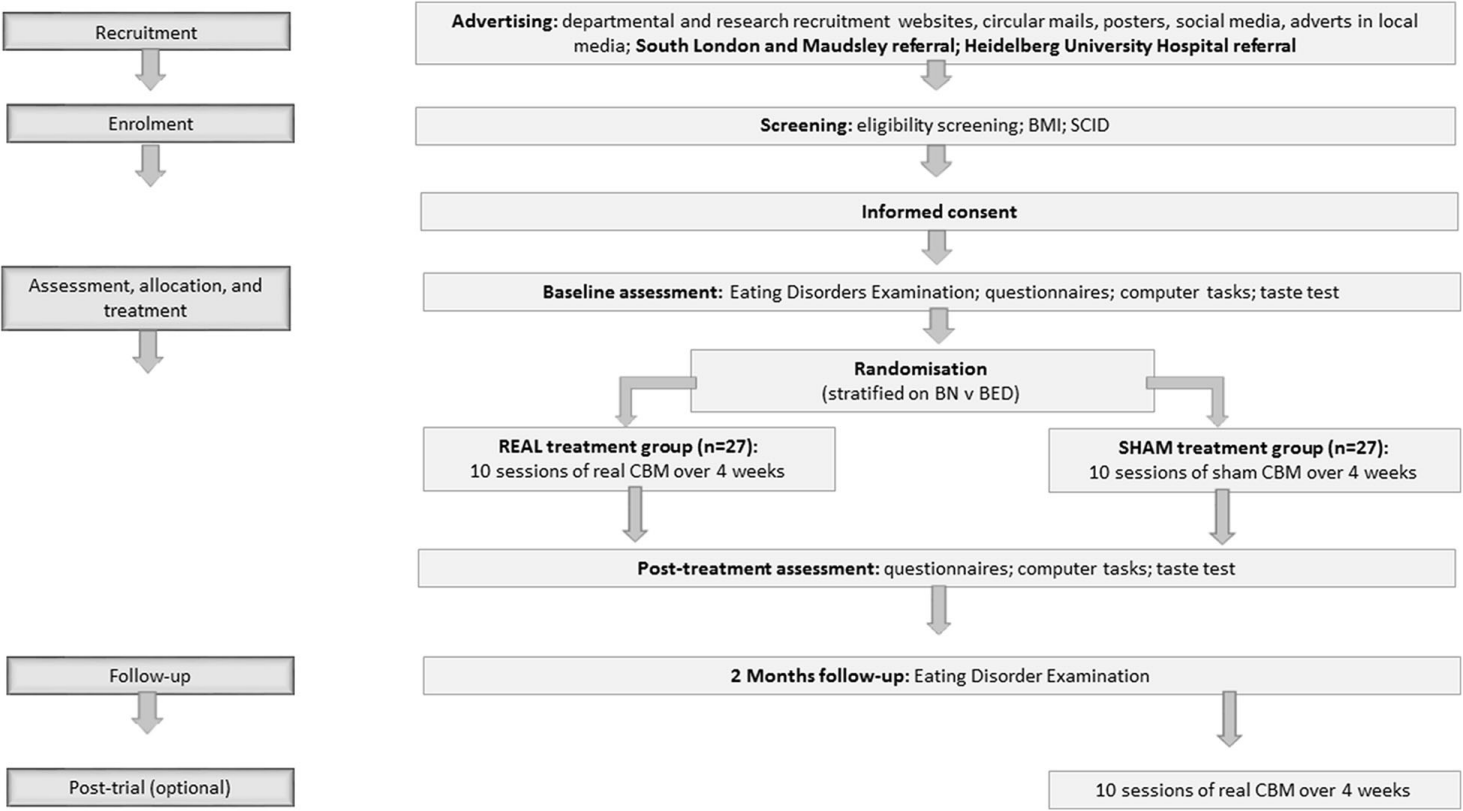
Participant flow chart

This is a superiority trial adopting a double-blind, randomised, placebo-controlled, parallel-group design comparing real and sham CBM. Participants with BN or BED will be randomly allocated to receive either 10 sessions (15 min each) of real CBM (treatment group) or sham CBM (control group) in a period of 4 weeks. Participants will be recruited from two different sites (Heidelberg, Germany and London, UK). Outcomes will be assessed at baseline, post-treatment and 2-month follow-up. The study protocol is outlined in the flow chart in Fig. 1, and Table 1 provides details of all assessments at the different timepoints.

**Table 1:** ABBA study schedule of enrolment, interventions and assessments

	Study period						
	Treatment (real CBM) group and control (sham CBM) group						Control group only (optional)
	Enrolment	Pre-allocation	Allocation	Treatment	Post- treatment		Treatment
Timepoint	0	Baseline assessment (t1)	0	Weeks 1 –4	Follow-up (t2)	2- month follow-up (t3)	Weeks 1–4
Enrolment:							
Eligibility screen	X						
Informed consent	X						
Allocation			X				
Interventions:							
Real CBM				10 x			10 x
Sham CBM				10 x			
Assessments:							
Weight	X						
Questionnaires		X			X		
Computer tasks		X			X		
Taste Test		X			X		
Clinical interview		X				X	

*CBM* cognitive bias modification

#### Participants and recruitment

Participants will be recruited from the outpatient units of the Department of General Internal Medicine and Psychosomatics at University Hospital Heidelberg, the South London and Maudsley NHS Foundation Trust’s Specialist Adult Eating Disorders Unit, and through websites, circular mails, advertising posters and advertisements in the local media.

### Inclusion criteria

Women and men will be eligible for participation if they are aged 18 years or above and meet the criteria of a *Diagnostic and Statistical Manual of Mental Disorders, version 5* (DSM-5) diagnosis of BN or BED [5].

### Exclusion criteria

Exclusion criteria are: (1) age under 18 years, (2) medical (e.g. electrolyte abnormalities) or psychiatric (e.g. acute suicidality) instability, (3) the need for immediate inpatient treatment, (4) lifetime diagnosis of substance dependence, psychosis, bipolar disorder, attention deficit hyperactivity disorder (ADHD) or borderline personality disorder, (5) psychotropic medication use other than selective serotonin reuptake inhibitors (patients have to be on a stable medication, i.e. at least 14 days of a SRRI during participation in the trial), (6) severe learning disability that affects patients’ ability to complete study assessments/treatment, and (7) the inability to speak fluent English/German (depending on study site), impacting on patients ability to complete study assessments/treatment.

### Sample size

Previous randomised controlled trials comparing real and sham versions of approach bias modification in clinical samples applied a repeated measures ANOVA design (group × time) to examine treatment-specific changes and have reported small-to-medium effect sizes (η_p_^2^ between 0.05 and 0.06) [27, 29, 30]. Using the smallest effect size that has previously been reported, a total sample size of 40 patients will have 80 % power to detect an effect of this size using a 2 × 2 repeated measures ANOVA with a 0.05 two-tailed significance level. The percentage of data lost owing to errors in the applied neuropsychological tasks was 7 % at the maximum in previous studies. Taking this and a potential dropout rate of 25 % into account, a minimum of 53 patients must be included. Thus, we will recruit 27 patients for each group (total *n* = 54).

### Intervention

Patients in the real CBM condition will conduct a treatment version of the Food Approach-Avoidance Task (Food-AAT) [39]. In this task, participants are shown colour photographs of food and control (i.e. neutral household and office) items on a computer screen. They are required to pull or push a joystick in response to the outline of the picture (round versus rectangular), irrespective of picture content. Format movement assignments are counterbalanced among participants (i.e. half will push round pictures and half will push rectangular pictures). When the joystick is pulled, the picture grows bigger and when it is pushed it grows smaller. This zooming-in and zooming-out emphasises respective sensations of approaching and avoiding and thus combines the proprioceptive (arm movement) and exteroceptive (zooming feature) cues of approach and avoidance behaviour [47]. The treatment version of the Food-AAT adopts an implicit learning paradigm by presenting all food pictures in the ‘push’ (i.e. avoid) format so that participants learn to link avoidance movements to visual cues of high-calorie food. Patients will receive ten 15-min sessions of training over a 4-week period.

Patients in the control condition (sham CBM) will receive the same dosage of the same task but will *not* be trained to avoid food cues in the Food-AAT. Instead, patients in the control condition receive 10 additional sessions of the pre- and post-treatment assessment version of the task (Food-AAT), which requires an equal number of approach and avoidance movements to both food and non-food pictures. Sham (placebo) CBM with an equal dosage, frequency and character was chosen as the comparator treatment in order to examine the specific effects of this form of CBM.

Sessions will take place in dedicated research facilities. In line with the vulnerability-stress model of cognitive biases described in the introduction [48], participants will be presented with a set of pictures that are considered to induce mild levels of negative mood at the beginning of every training session (60 images; duration: approximately 1 min) [49].

Study adherence: a researcher will be present throughout the training and assessment sessions to ensure study adherence. The interventions are predefined, computerised tasks. Any deviation from the study protocol will be recognised and recorded immediately. We will ask participants to refrain from any other treatment that focuses on their eating disorder. However, if clinical need dictates another treatment, this will be documented but will not lead to the exclusion of the participant. Participants are allowed to discontinue the treatment at any time.

### Procedure

A flowchart outlining the study procedures is presented in Fig. 1. For further information about the time schedule of enrolment, interventions and study assessments, please see Table 1.

#### Screening

Potential participants will be referred to the study by their clinician or by themselves. Study researchers will screen participants for eligibility. Screening includes the Structured Clinical Interview for DSM Disorders (SCID) [50–52], a short inclusion/exclusion screen specific to this study, and an assessment of medical and psychiatric history and medication dosage and stability. In line with the Consolidated Standards of Reporting Trials (CONSORT) guidelines, we will record the number and reasons for any participants we must exclude, or any who decline consent or withdraw from the study.

#### Baseline assessment

Binge-eating frequency and general eating disorder symptomatology will be assessed in terms of a specific, structured clinical interview, i.e. the Eating Disorders Examination [53, 54]. Participants will also be weighed and asked to complete a battery of questionnaires relating to eating disorder symptomatology, food craving, depression and anxiety, as well as neuropsychological computer tasks that assess approach bias and attentional bias towards visual food cues and working memory. Furthermore, state levels of food craving after cue exposure and food intake will be assessed in laboratory tasks.

Once the baseline assessment is completed, participants will be randomly allocated to the treatment (real CBM) or control (sham CBM) group. Randomisation will be performed independently from the trial team by a central study office at Heidelberg University Hospital. The first CBM session will start on the day of the baseline assessment. Participants in the sham group will be offered the opportunity to receive real CBM after the end of follow-up.

#### Post-treatment assessment

The post-treatment assessment will take place after the last training session, and will include the same elements as the baseline assessment except for the Eating Disorders Examination.

#### Two-month follow-up

Two months after the post-treatment assessment a follow-up session will be conducted which will involve the Eating Disorders Examination. Finally, the success of blinding will be evaluated by asking participants to guess the treatment allocation. Participants will be unmasked and individuals in the control group will then be offered the real treatment.

### Measures

#### Screening measures

- ▪ Body Mass Index (kg/m^2^)
- ▪ Screening module of the SCID [50–52]: this semistructured psychiatric interview will be used as a diagnostic screen to assess the presence of psychiatric comorbidities.
- ▪ The depression module of the Patient Health Questionnaire (PHQ-9) [55, 56] and the General Anxiety Disorder Screener-7 (GAD-7) [57, 58] are reliable and valid as well as widely used self-report instruments to assess symptoms of depression and anxiety according to the DSM-5.

#### Primary outcome measure

- ▪ Eating Disorder Examination (EDE) [53, 54]: the primary outcome variable in this study is the treatment-specific change from baseline to 2-month follow-up in the number of objective binge-eating episodes as assessed by the EDE. The EDE is a reliable and valid as well as widely used measure of eating disorder symptoms and is regarded as the instrument of choice for the assessment and diagnosis of eating disorders according to the DSM-5.

#### Secondary outcome measures

- ▪ Food Approach-Avoidance Task (Food-AAT), assessment version [16, 39, 59]: this task will be used to assess approach bias towards visual cues of high-calorie food (expected target cognitive mechanism of intervention). It is identical to the treatment version except that in the assessment version of the task, the required response is unrelated to the picture content (i.e. food and neutral stimuli are presented equally often in round and rectangular, i.e. push and pull, format). The approach bias towards food is calculated by subtracting the median reaction time (RT) for pulling food pictures from the median RT for pushing pictures of this category [59]. Thus, a positive value denotes an approach bias (i.e. the participant is faster in pulling than in pushing food pictures), whereas a negative value indicates an avoidance bias.
- ▪ Visual probe task [60]: this task will be used to assess participants’ attentional bias towards visual food cues. During this computer task picture pairs (food/non-food) are presented side by side on a computer screen after the presentation of a fixation point at the centre of the screen and are followed by the presentation of a probe (denoted by *) appearing in the location of one of the pictures. Participants are instructed to indicate the location of the probe by pressing a corresponding key on a keyboard as quickly as possible. The position of the food and neutral pictures are balanced across trials so that each appears in either location with equal frequency. Recordings of the participant’s manual response latency when indicating the location of the probe are used to calculate a response latency bias. The logic of this task presumes that participants who have an attention bias towards food are quicker in responding to indicate the location of the probe when the probe replaces a food stimulus. Thus, the latencies of manual responses provide an index for attention bias for food. Positive values indicate faster mean latencies (i.e. an attentional bias) to food cues than for neutral cues.
- ▪ The Food Challenge Task [16, 39, 61]: the Food Challenge Task will be used to examine cue-induced food craving. Participants rate their state levels of food craving using the Food Cravings Questionnaire State Version [62, 63] after being presented with filmed cues of highly appetitive foods.
- ▪ Taste Test: food intake will be measured by means of a ‘Taste Test’. Participants will be instructed to rate three bowls of highly palatable food items (chocolate, crisps, fruit gums) in terms of their visual attractiveness, smell and taste. The participants will be told that they are free to try as much of the offered items as they like. Consumption will be determined by weighing the bowls both before and after the Taste Test.
- ▪ The digit-span forward and backward subtests of the Wechsler Memory Scale III [64] will be used to measure working memory capacity.
- ▪ Global eating disorder psychopathology will be assessed by the EDE [53, 54].
- ▪ The Food Cravings Questionnaire Trait Version [62, 63]: this is a reliable and valid self-report questionnaire that measures trait levels of craving for food.
- ▪ Treatment acceptance will be assessed using a feedback form composed of several standard questions regarding the acceptance of the intervention [39].

Confidentiality and anonymity of all personal data will be retained throughout the entire study. Manual files will be securely locked in a lockable filing cabinet and all electronic files will be password-protected. Identifying information will be removed from the data, stored separately and replaced with a numeric identification code. The master list of names which correspond to each participant’s numeric identification code will be stored electronically and will be password-protected. This information will only be accessible to key researchers involved in the study.

### Randomisation

Randomisation will be performed independently from the trial team by a central study office at Heidelberg University Hospital using a specific randomisation software (RANDI2). Randomisation will be stratified for BN and BED in a 1:1 ratio. Participants in the sham group will be offered the opportunity to receive real CBM after the end of follow-up.

### Blinding

Participants and assessors using the EDE will be blinded to treatment allocation throughout, i.e. the study will be conducted in a double-blind fashion. To assess whether allocation concealment has been successful, participants will be asked to guess the treatment allocation at the end of treatment and to indicate how certain they are of this guess. Participants will be debriefed and unblinded to group allocation upon completion of the 2-month follow-up.

### Analyses

To determine quality, completeness and variability of the outcome measures, descriptive statistical analyses and graphical methods will be used. To evaluate treatment-specific changes over time in patients, a 2 × 2 repeated measures ANOVA, with group as between-subject factor and time as within-subject factor, will be applied. The size of the treatment effect on the outcome measure (EDE) will be the difference in outcome data between those in the two treatment conditions at follow-up. Additionally, multiple regression models will permit exploration of the relationship between changes in the cognitive measures and symptom improvement. No interim analyses are planned. A two-tailed significance level of *p* < .05 will be used throughout. Both completer and intent-to-treat analyses will be performed. Missing values will be replaced by using the Last Observation Carried Forward (LOCF) method for the intent-to-treat analysis. As this is a pilot trial resources do not stretch to setting up a Monitoring Committee. Analyses will be conducted by the principal investigators. As CBM is a safe procedure with no known side effects we do not expect any unexpected adverse reactions, serious or unexpected serious adverse reactions. We will report any adverse reactions and serious adverse events. It is intended that the results of the study will be reported and disseminated by the principal investigators at international conferences and in peer-reviewed scientific journals.

## Discussion

The ABBA study aims to examine the potential of a novel, computerised, short-term treatment module for patients with bulimic eating disorders that targets a specific cognitive bias in the processing of visual food cues, i.e. automatic approach tendencies towards highly palatable food. This is the first study examining this treatment approach in a clinical sample of patients with BN and BED. Particularly in view of the high attrition and relapse rates in bulimic eating disorders, novel treatment approaches are urgently needed [4, 65]. Strengths of the presented study include a rigorous, double-blind, randomised controlled design including a strong control condition with a similar task and equal dosage, and the assessment of potential action mechanisms. Several potential practical and operational issues, however, may pose challenges to the successful and timely completion of the study, particularly regarding recruitment and attrition. Patients with eating disorders are often ambivalent about treatment, which is reflected in high attrition rates and this may become particularly difficult if a participant believes that they are receiving sham treatment, or if the treatment and assessments are experienced as too burdensome.

### Trial status

Participant recruitment and data collection have already begun by the time of manuscript submission.

## Abbreviations

AAT: Approach-Avoidance Task
BED: Binge eating disorder
BN: Bulimia nervosa
CBM: Cognitive bias modification
CONSORT: Consolidated Standards of Reporting Trials
DRKS: Deutsches Register Klinischer Studien (German Clinical Trials Register)
DSM: Diagnostic and Statistical Manual of Mental Disorders
EDE: Eating Disorder Examination
ICMJE: International Committee of Medical Journal Editors
WHO: World Health Organisation
